# Expression of VEGF Receptors on Endothelial Cells in Mouse Skeletal Muscle

**DOI:** 10.1371/journal.pone.0044791

**Published:** 2012-09-12

**Authors:** Princess I. Imoukhuede, Aleksander S. Popel

**Affiliations:** 1 Department of Bioengineering, University of Illinois Urbana Champaign, Urbana, Illinois, United States of America; 2 Department of Biomedical Engineering, School of Medicine, Johns Hopkins University, Baltimore, Maryland, United States of America; Bristol Heart Institute, University of Bristol, United Kingdom

## Abstract

VEGFR surface localization plays a critical role in converting extracellular VEGF signaling towards angiogenic outcomes, and the quantitative characterization of these parameters is critical for advancing computational models; however the levels of these receptors on blood vessels is currently unknown. Therefore our aim is to quantitatively determine the VEGFR localization on endothelial cells from mouse hindlimb skeletal muscles. We contextualize this VEGFR quantification through comparison to VEGFR-levels on cells in vitro. Using quantitative fluorescence we measure and compare the levels of VEGFR1 and VEGFR2 on endothelial cells isolated from C57BL/6 and BALB/c gastrocnemius and tibialis anterior hindlimb muscles. Fluorescence measurements are calibrated using beads with known numbers of phycoerythrin molecules. The data show a 2-fold higher VEGFR1 surface localization relative to VEGFR2 with 2,000–3,700 VEGFR1/endothelial cell and 1,300–2,000 VEGFR2/endothelial cell. We determine that endothelial cells from the highly glycolytic muscle, tibialis anterior, contain 30% higher number of VEGFR1 surface receptors than gastrocnemius; BALB/c mice display ∼17% higher number of VEGFR1 than C57BL/6. When we compare these results to mouse fibroblasts in vitro, we observe high levels of VEGFR1 (35,800/cell) and very low levels of VEGFR2 (700/cell), while in human endothelial cells in vitro, we observe that the balance of VEGFRs is inverted, with higher levels VEGFR2 (5,800/cell) and lower levels of VEGFR1 (1,800/cell). Our studies also reveal significant cell-to-cell heterogeneity in receptor expression, and the quantification of these dissimilarities ex vivo for the first time provides insight into the balance of anti-angiogenic or modulatory (VEGFR1) and pro-angiogenic (VEGFR2) signaling.

## Introduction

The vascular endothelial growth factors (VEGF) are key factors involved in angiogenesis, the growth of new blood vessels from existing blood vessels. Under conditions of hypoxia, VEGF is upregulated in parenchymal and stromal cells by the binding of the transcription factor, HIF1α, to the VEGF gene promoter [1]. Once secreted by these cells, VEGF binds to its receptors on endothelial cells. VEGF binding activates cell signaling resulting in the endothelial cell proliferation and migration necessary for angiogenesis. Understanding how ligand-receptor binding progresses towards angiogenesis is complicated by the fact that VEGF receptor 1 (VEGFR1) exhibits both pro-angiogenic and anti-angiogenic properties. VEGFR1 may serve as a positive regulator under pathological conditions, where its expression may promote angiogenesis [2]. VEGFR1 may also serve as a negative regulator both through downregulation of VEGFR2-mediated signaling [3] and due to its 10-fold higher-affinity for VEGF, compared to VEGFR2, but low tyrosine kinase activity [4], [5].

Systems biology offers promising approaches to predict how VEGF-VEGFR interactions correlate with either pro-angiogenic or anti-angiogenic signaling outcomes. Recent computational models, based on mass-action kinetics, have focused on VEGF-VEGFR binding, given the role of this signaling axis as a mediator and biomarker of pathological angiogenesis [6], [7], [8]. These computational models have predicted the distribution of VEGF within diseased tissue, healthy tissue, and blood, and the effect of anti-VEGF therapeutics on ligand concentrations [9], [10]. Additionally, models have predicted the dependence of heterodimerization (VEGFR1/2) and homodimerization (VEGFR1/1 or VEGFR2/2) on receptor expression, specifically when levels of VEGFR1 and VEGFR2 vary, the proportion of dimerized receptors can shift towards either a preponderance of pro-angiogenic VEGFR2 homodimers or dominance by anti-angiogenic or modulatory VEGFR1 homodimers [11]. Therefore, determining absolute numbers of these receptors ex vivo should provide insight into the angiogenic signaling balance.

Previous quantification of VEGFR reported surface-levels 500–50,000 VEGFR1/cell and 6,000–150,000 VEGFR2/cell; these variations can be attributed to the use of non-human, clonal, and transfected cells [12], [13], [14], [15], while Scatchard analysis on HUVECs has previously reported 4,200 VEGFR1/HUVEC and 12,400 VEGFR2/HUVEC [16]. Recent quantitative fluorescence cytometry performed in our laboratory has determined the levels of VEGFR1, VEGFR2, VEGFR3 and NRP1 on human umbilical vein endothelial cells, human dermal microvascular endothelial cells, and human dermal lymphatic microvascular endothelial cells [17]. Our studies revealed similarity in the order of magnitude of VEGFR1 and VEGFR2 density in vitro; however given the greater structural and molecular complexity within tissue, we expect that VEGFRs may display differential expression patterns ex vivo compared to within cell culture. Thus, we aim to quantify VEGFR levels on endothelial cells isolated from skeletal muscle and compare these results to cultured, in vitro cells.

Our quantification of VEGFRs involves the use of two mouse strains: C57Bl/6 and BALB/c, since mouse strains can exhibit different vascular characteristics and response to vascular injury [18], [19], [20], [21], [22], [23], [24], [25]. Recent imaging studies of C57Bl/6 and BALB/c skeletal muscle arteriolar networks (spinotrapezious, latissimus dorsi, and thoracic diaphragm), have identified significantly different structure in arteriole-to-arteriole linkages between these mouse strains with C57Bl/6 mice exhibiting arcaded arteriolar trees and BALB/c mice displaying a dendritic structure [25]. Furthermore, following hindlimb ischemia, C57BL/6 mice express 2–6–fold higher VEGF-A_120/164/188_ than BALB/c mice [19]. BALB/c mice also display lower perfusion recovery and greater tissue loss than C57BL/6 mice [26], [27], following ischemia, and these variations have been mapped to mouse LSq-1 locus [21]. We hypothesize that these structural, genetic, and ligand- level differences, may translate to differential VEGFR densities on endothelial cells across these strains.

VEGFRs in microvessels adjacent to muscle fibers of different types may also show differential expression patterns, in part because differential VEGF protein expression has been previously observed in oxidative and glycolytic muscle [28]. This phenomenon has also been observed at the level of VEGF mRNA, with higher VEGF expression in oxidative muscle [29], [30]. Therefore we hypothesize that VEGFRs may be differentially presented across muscle types and we measure receptor levels in tibialis anterior, a highly glycolytic muscle, and gastrocnemius, a mixed muscle (containing both glycolytic and oxidative fibers).

Stochasticity in gene expression leads to cell-to-cell variability in mRNA and protein levels, which can translate to heterogeneous receptor levels across similar endothelial cells [31], [32], [33]. Furthermore, within tissue, endothelial cells can take on differing roles (e.g. endothelial cells involved in sprouting angiogenesis can take on either primarily proliferative phenotype of stalk cells or primarily migrating phenotype of tip cells); these differing endothelial cell phenotypes may contribute to innate heterogeneity in surface-receptor levels. To quantify endothelial cell heterogeneity on the receptor level, we characterize receptor localization on a cell-by-cell level [34], [35]. Additionally, we define receptor heterogeneity in the context of endothelial cells and fibroblasts in vitro. The ex vivo data for the first time, define the range of VEGFRs expressed at the angioquiescent state and provide insight into the balance of angiogenic receptors in murine tissue.

## Methods

### Ethics

The studies in the article took place at The Johns Hopkins University and as such, the animals were housed in approved animal facilities at The Johns Hopkins University School of Medicine in compliance with state and federal laws and policies that govern use of animals in research. The National Research Council’s Guide to the Care and Use of Laboratory Animals served as the primary source for standards for the Johns Hopkins Animal Care and Use Program. Johns Hopkins University met the requirements of the Animal Welfare Act (AWA) and Public Health Service (PHS) policy. The Animal Resources were accredited by the Association for the Assessment and Accreditation of Laboratory Animal Care (AAALAC) and staffed by full-time veterinarians and certified animal care workers. All of the protocols used were approved by the Institutional Animal Care and Use Committee at Johns Hopkins University.

### Cell Culture

Human umbilical vein endothelial cells (HUVEC), human dermal microvascular endothelial cells (MEC), and human dermal lymphatic microvascular endothelial cells (LEC) were acquired from individual donors (Lonza, Walkersville, MD and Stem Cell Technologies, Vancouver, Canada). The endothelial cells were maintained in Endothelial Cell Growth Medium-2 (EGM-2), supplemented by the EGM-2 SingleQuot Kit for HUVECs, or supplemented by the EGM-2 Microvascular SingleQuot Kit for MECs. Mouse fibroblasts (BALB/3T3 clone A31) were acquired from American Type Culture Collection and were maintained in DMEM containing 10% fetal bovine serum (Invitrogen) and 1% Penicillin-Streptomycin (Invitrogen). Cells were grown at 37°C in 95% air, 5% CO_2_. Cells were grown to confluence before dissociating and cells were only used up to passage 6. For routine cell culture, cells were detached from flasks using 0.25% trypsin (Invitrogen, Carlsbad, CA); for quantitative flow cytometry experiments, the non-enzymatic, cell dissociation solution (Millipore, Billerica, MA) was applied for 5–7 minutes at 37°C. Cells were resuspended in 10 mL cold FBS stain buffer (BD Biosciences, San Jose, CA), centrifuged at 300×g for 4 minutes, supernatant was aspirated, and cells were resuspended in 10 ml cold FBS stain buffer. Cells were centrifuged and resuspended to a final concentration of 4×10^6^ cells/mL in cold FBS stain buffer.

### Endothelial Cell Isolation

Mice were euthanized with CO_2_ for ∼5 minutes. Gastrocnemius and tibialis anterior were extracted from male and female 8–14 week old C57BL/6 (Charles River and NCI) and BALB/c (NCI) mice, with a mean weight of 22.4±0.4 g (∼20 minutes). Excised tissue was placed in a 50 mL conical tube containing HBSS without calcium and without magnesium (Mediatech, Manassas, VA). Muscle tissue was sectioned on an ice block, as previously described [36], [37], [38]. Briefly, tissue was minced into 1 mm sections and added to freshly prepared, cold, 0.2% collagenase type 4, filtered (Worthington Biochemical Corporation, Lakewood, NJ), which had been reconstituted in HBSS without calcium and without magnesium (∼20 minutes). Muscle tissue was digested for 30 min at 37°C with vortexing every 10 minutes. The enzymatic reaction was stopped by adding 2 parts Isolation Buffer, containing PBS without calcium and magnesium (Invitrogen), 2 mM EDTA (Mediatech), and 0.1% BSA (Sigma). The tissue mixture was passed through a 70 µm strainer (BD). Cells were centrifuged at 300×g for 5 minutes at 4°C and resuspended in 30 mL cold isolation buffer. Figure S1 displays the flow cytometry traces from staining this mixed suspension of cells with anti-CD34-FITC and anti-VEGFR2-PE. The CD34^+^/VEGFR2^+^ cell yield was 5% of the total cell suspension. Endothelial cells were isolated from the cell suspension using DSB-X (Invitrogen) biotinylated mouse CD31 antibody (eBioscience and BD Bioscience, San Diego, CA) and FlowComp Dynabeads (Invitrogen) according to the manufacturers’ instructions (total time ∼ 4 hours).

### Cell Staining and Flow Cytometry

25 µL aliquots of isolated cells (∼1×10^4^ cells) were added to tubes and were dually labeled with 10 µL of fluorescein isothiocyanate-conjugated monoclonal antibody to mouse CD34 (BD Pharmingen) and phycoerythrin (PE)-conjugated monoclonal antibody at a final concentration of 14 µg/mL for VEGFR1 and VEGFR2 (R&D). Since CD31 is also expressed on T cells, B cells, NK cells, macrophages/monocytes, granulocytes, and platelets, we employ CD34-FITC as a secondary marker, which is expressed on endothelial cells, stem cells/precursors, mast cells, and neurons, the latter of which should be excluded by the prior CD31 magnetic bead separation [39], [40]. The concentrations were reported to be saturating by the manufacturer, and we previously used anti-hVEGFR1-PE, anti-hVEGFR2-PE, anti-hVEGFR3-PE, and anti-hNRP1-PE (R&D Systems) at concentrations recommended by the manufacturer and independently confirmed those concentrations to be saturating [17]. Tubes were protected from light and incubated for 40 minutes on ice. Cells were washed, centrifuged twice with 4 mL FBS stain buffer, and resuspended in 400 µL stain buffer.

The precision and accuracy of quantitative flow cytometry has been rigorously tested [41], [42], [43]. We chose the phycoerythrin (PE) fluorophore as the basis of our quantitative fluorescence measurements, because its high extinction coefficient reduces error due to photobleaching and its size minimizes the possibility of multiple fluorophores conjugated to an antibody. Furthermore, we have previously applied FRET to confirm that the antibodies can recognize and bind to dimerized receptors [17].

As previously described, flow cytometry was performed on either a FACSCalibur or a FACScan; CellQuest (BD) software was used for data acquisition and Flow Jo (TreeStar) was used for data analysis [17]. Tubes were vortexed immediately prior to placement in the flow cytometer. 5,000–10,000 cells were collected. Single cells and Quantibrite phycoerythrin beads (BD Biosciences) were selected (gated) using linear side scatter and forward scatter plots (Figure S2). CD34-FITC-positive cells (FL1 channel) were further gated to select the endothelial cell population (Figures S3,S4,S5). Histograms were used to determine phycoerythrin (FL2-channel) geometric means for the Quantibrite phycoerythrin beads using the same compensation and voltage settings for acquiring cell fluorescence data (Figure S2D & S6A). Using the phycoerythrin geometric means, and the number of phycoerythrin molecules/bead for fluorescence values of low (515 phycoerythrin molecules/bead), medium-low (5,956 phycoerythrin molecules/bead), medium-high (26,653 phycoerythrin-molecules/bead), and high (69,045 phycoerythrin-molecules/bead) fluorescing beads provided by BD, a calibration curve was formed, which was fitted by a linear regression: y = mx+b where “x” represents log_10_(phycoerythrin molecules/cell), “y” represents log_10_(FL2 geometric mean for anti-VEGFR-phycoerythrin), “m” represents the slope of phycoerythrin-bead calibration curve, and “b” represents the y-intercept of the phycoerythrin-bead calibration curve (Figure S6B). The phycoerythrin geometric means from antibody-labeled cells were used to determine the number of receptors bound per cell. Non-labeled cells were imaged to determine endogenous fluorescence and the corresponding number of phycoerythrin molecules/cell. This value was subtracted from the number of receptors bound per cell.

### Cell-by-Cell Analysis

For a given experiment, single-cell fluorescence intensity data from the gated population was extracted using FlowJo (Tree Star, Ashland, OR). Endothelial cell phycoerythrin fluorescence intensity was converted to number of receptors per cell using the phycoerythrin bead calibration obtained during that imaging session. Data on the number of receptors per cell were pooled and any data greater than 3 standard deviations from the mean were excluded. Histograms were created with bins of 500 receptors/cell. Median and coefficient of variation are reported in Table 1. A two-sample Kolmogorov-Smirnov (K-S) test was performed in Matlab (MathWorks, Natick, MA) to determine whether histograms were from a common distribution. In each case, the K-S test found the distributions to be significantly different.

**Table 1:** **Surface receptor statistics.**

	Sample	Ensemble averaging		Cell by cell analysis				
		n	Mean ± SEM	Number of cells	Median	Coefficient of Variation	Skewness	Kurtosis
**VEGFR1**	BALB/c							
	Gastrocnemius	6	2,600±400	20,149	2,500	830%	25	630
	Tibialis Anterior	6	3,700±500	10,039	3,300	740%	15	220
	C57BL/6							
	Gastrocnemius	11	2,000±200	33,795	1,800	110%	4.8	41
	Tibialis Anterior	10	3,200±200	14,740	3,000	120%	4.5	35
	Compiled Mouse ECs	33	2,800±200	78,723	2,300	780%	31	1000
	Human ECs (in vitro)	63	1,800±100	149,124	2,200	709%	180	42,000
	Fibroblasts/3T3 (in vitro)	9	35,800±5,200	88,538	31,800	91%	1.7	2.6
**VEGFR2**	BALB/c							
	Gastrocnemius	6	1,600±400	26,065	1,800	480%	53	3500
	Tibialis Anterior	6	2,000±400	14,020	2,000	990%	23	520
	C57BL/6							
	Gastrocnemius	11	1,300±100	41,741	1,300	100%	5.3	67
	Tibialis Anterior	10	1,700±200	25,799	2,000	95%	5.9	96
	Compiled Mouse ECs	33	1,600±100	107,625	1,600	750%	56	3300
	Human ECs (in vitro)	58	5,800±300	141,513	7,300	485%	74	7800
	Fibroblasts/3T3 (in vitro)	9	700±100	90,482	2,000	69%	10	180

Endothelial cells from gastrocnemius (mixed muscle) and tibialis anterior (white/glycolytic muscle) were isolated from 31 C57BL/6 and 20 BALB/c mice. 3T3 fibroblasts, obtained from ATCC, represent a mouse in vitro sample.

### Statistical Analysis

Values are expressed as mean ± standard error of the mean. Unless otherwise noted, p<0.05 is considered statistically significant using the analysis of variance (ANOVA) Tukey test.

## Results

### Optimizing Quantification ex vivo

**Figure 1:**
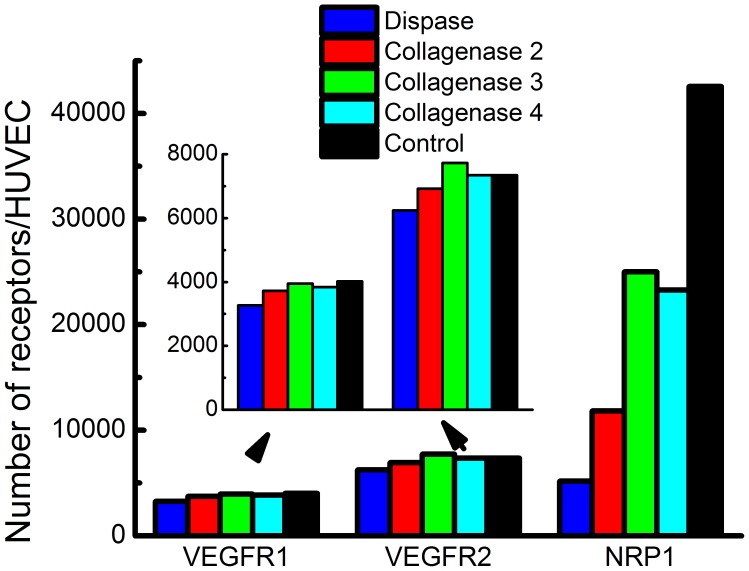
Dissociation enzymes. 0.2% collagenase or 0.2% dispase was applied to HUVECs for 30 min at 37°C with intermittent vortexing to determine whether VEGFR1, VEGFR2, and NRP1 surface levels are sensitive to dissociation enzymes. Collagenase 4 did not affect VEGFR1 or VEGFR2 density. Each enzyme affected NRP1 density.

Our previous quantification of VEGFR density in vitro determined conditions for performing receptor quantification. We confirmed antibody specificity by testing porcine aortic endothelial (PAE) cells (kindly provided by Dr. Shay Soker, Wake Forest University) expressing VEGFR2 and NRP1, identified saturating antibody labeling concentration, ensured preservation of target receptors by testing cell-dissociation conditions, and established monomeric antibody binding through flow cytometry-FRET experiments [17]. Additional steps are required for ex vivo quantification, including: tissue harvest and tissue dissociation; to determine whether receptor levels are compromised during enzymatic digestion, we tested HUVECs under the enzymatic dissociation conditions used on the skeletal muscle tissue. We incubated HUVECs for 30 min at 37°C with vortexing every 10 minutes, in the following dissociation enzymes: collagenase 2, 3, 4, and dispase. We determined that VEGFR1 and VEGFR2 surface-levels are unaffected by collagenase digestion in the HUVECs. This is a significant finding, since serine proteases (e.g. trypsin), intrinsic to collagenases, can significantly affect cell isolation yields [44]. This finding mirrors our previous in vitro work showing VEGFR1 and VEGFR2 surface-levels being unaffected by trypsin [17]. However, NRP1 surface-levels on the HUVECs are significantly affected by each of the enzymes tested (Figure 1). Again, serine proteases may partially account for this enzyme-induced decrease in NRP1 surface density. The collagenase-mediated decrease in NRP1 levels is consistent with our previous in vitro finding that trypsin significantly decreases NRP1 levels [17]. As such, we do not report ex vivo quantification of the NRP1 co-receptor.

### Ensemble Analysis of VEGFR Localization

**Figure 2:**
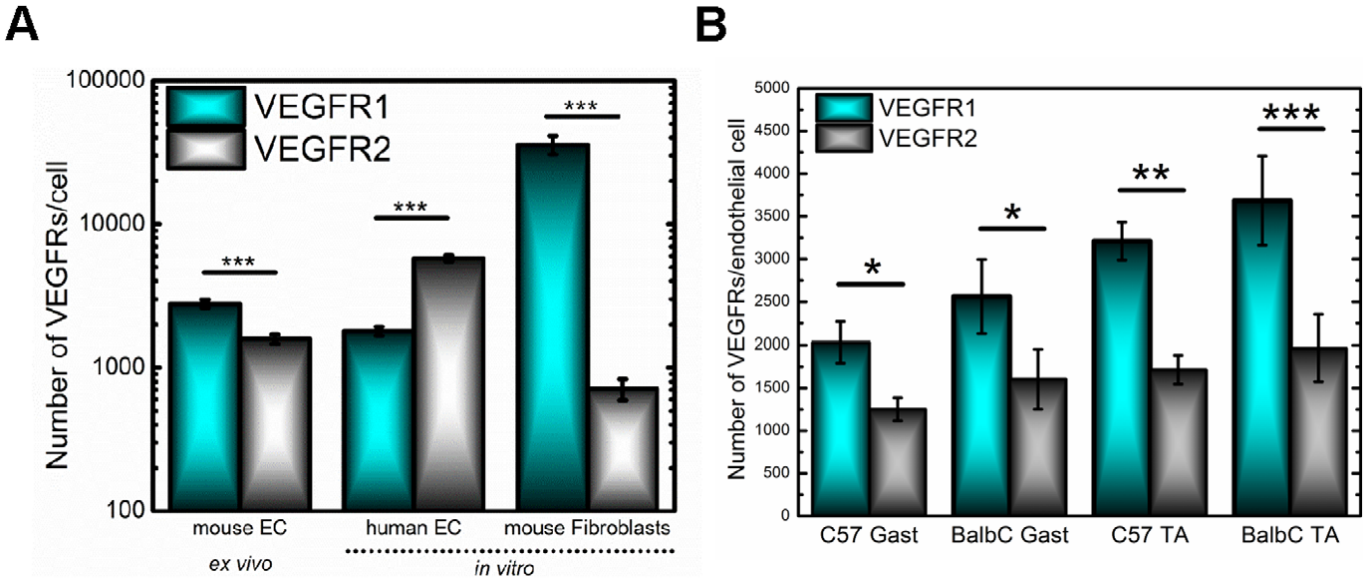
Cell surface expression of VEGFR1 and VEGFR2. (A) Receptor levels from mouse ex vivo endothelial cells, human in vitro endothelial cells and mouse in vitro fibroblasts are plotted on a log-scale. Mouse endothelial cells freshly isolated from skeletal muscle have an average surface expression of 2,800 VEGFR1/endothelial cell and 1,600 VEGFR2/endothelial cell, representing significantly higher VEGFR1 relative to VEGFR2 (p<0.001). Cultured, human endothelial cells display 1,800 VEGFR1/endothelial cell and 5,800 VEGFR2/endothelial cell, a significant difference in surface expression (p<0.001). The fibroblasts have an average surface expression of 36,000 VEGFR1/fibroblast and 700 VEGFR2/fibroblast, representing significant differences between VEGFR1 and VEGFR2 surface levels (p<0.001). (B) Endothelial cells from C57BL/6 have an average endothelial surface expression of 2,000 VEGFR1/cell and 1,300 VEGFR2/cell within the gastrocnemius and 3,200 VEGFR1/cell and 1,700 VEGFR2/cell within the tibialis anterior. BALB/c have an average endothelial surface expression of 2,600 VEGFR1/cell and 1,600 VEGFR2/cell within the gastrocnemius and 3,700 VEGFR1/cell and 2,000 VEGFR2/cell within the tibialis anterior.

The isolated mouse endothelial cells display significantly higher levels of VEGFR1 (2,800±200 receptors/cell) compared to VEGFR2 (1,600±100 receptors/cell) (p<0.001), representing a nearly 2-fold difference in VEGFR1: VEGFR2 surface-levels (Figure 2A). We also analyze VEGFR levels on commonly used in vitro cells, human endothelial cells (HUVECs, MECs, and LECs). When we compare the surface-receptor levels, we see that the receptor balance is inverted, with a significantly higher VEGFR2 surface expression in vitro (5,800±300 receptors/cell) compared to VEGFR1 (1,800±100 receptors/cell), which constitutes a greater than 3-fold difference in VEGFR1: VEGFR2 surface-levels. To determine whether the high ex vivo VEGFR1 surface expression is unique to ex vivo cells, we examine VEGFR1 and VEGFR2 localization on a BALB/c 3T3 (fibroblast) cell line (Figure 2A): fibroblasts being a readily available cell line, which secrete the angiogenic factors necessary for microvascular patterning and stabilization [45], [46]. The high-VEGFR1, low-VEGFR2 balance seen on the mouse skeletal muscle endothelial cells is also present on these fibroblasts; however, we observe a 50-fold higher level of VEGFR1 versus VEGFR2 with 36,000 VEGFR1/fibroblast and 700 VEGFR2/fibroblast.

We investigate two mouse strains, C57BL/6 and BALB/c with known differences in VEGF localization, vascular density, and responses to ischemia to determine if differential receptor surface density is also observed [19], [21], [28]. We measure a slightly higher (∼17%) VEGFR1 localization on BALB/c endothelial cells as compared to C57BL/6 endothelial cells; however, an analysis of variance (ANOVA) of these ensemble averaged data does not deem this difference as significant (Figure 2B and Table 1). To determine whether different muscle tissue presents distinct VEGFR levels we examine both tibialis anterior, which comprises mostly glycolytic fibers and gastrocnemius muscle, which is a mixed muscle tissue, containing both glycolytic and oxidative fibers. (We do not present a more-purely oxidative tissue, such as the soleus, because its small size does not allow for sufficient yield of endothelial cells–data not shown). The data show that surface-localized VEGFR1 is more numerous on the tibialis anterior compared to the gastrocnemius in each mouse strain. These correspond to 31% and 37% higher VEGFR1 densities in the tibialis anterior from BALB/c mice and C57BL/6 mice, respectively; however, as with the strain comparison, an ANOVA on the averaged data did not reveal these differences to be significant. We also examine male versus female mice and determine that overall no significant sex differences are observed between males and female mice; however, in tibialis anterior of C57BL/6 mice, we do observe significantly higher VEGFR2 levels on male endothelial cells compared to female endothelial cells (Figure S7).

### Cell-to-cell Variability in VEGFR Localization

Cell-by-cell analysis of VEGFR levels reveals that receptor surface distributions are non-Gaussian as determined by a Kolmogorov-Smirnov (K-S) test (p<0.001). Despite the non-normality, analyses of variation from the mean provide insight on the variability of receptor localization across endothelial cells, and we determine that endothelial cells exhibit significant heterogeneity in surface localization, represented by the high coefficient of variation in Table 1 for each condition. We also analyzed the skewness and kurtosis to quantitatively describe the distributions relative to a Gaussian distribution. The positive skewness for each of the distributions, represents the decreasing frequency of endothelial cells with high-surface VEGFRs [47], while the positive kurtosis describes the heavier tails and higher peak of the distributions relative to a normal distribution [48], [49].

**Figure 3:**
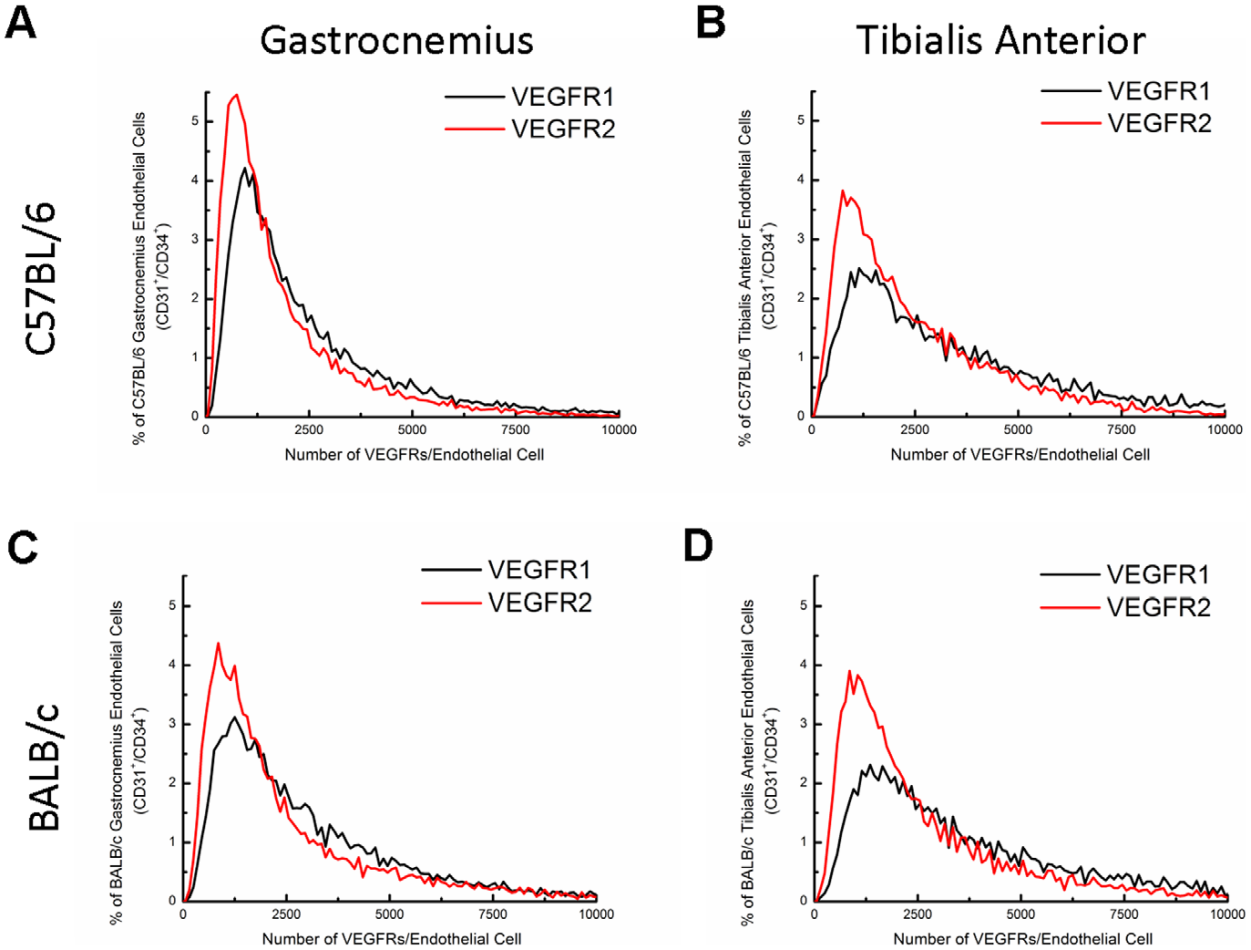
Cell-by-cell analysis of VEGFR1 and VEGFR2 distribution on mouse endothelial cells. The distributions show that there is significant heterogeneity in endothelial surface expression of VEGFRs, with 90% of endothelial cells expressing up to 10,000 receptors. The distributions also reveal a significant population of endothelial cells expressing low numbers of VEGFR2 (≤2,500 receptors/cell), thus lowering the average number of VEGFR2/cell relative to VEGFR1. (A) C57BL/6 gastrocnemius, (B) C57BL/6 tibialis anterior, (C) BALB/c gastrocnemius, (D) BALB/c tibialis anterior.

**Figure 4:**
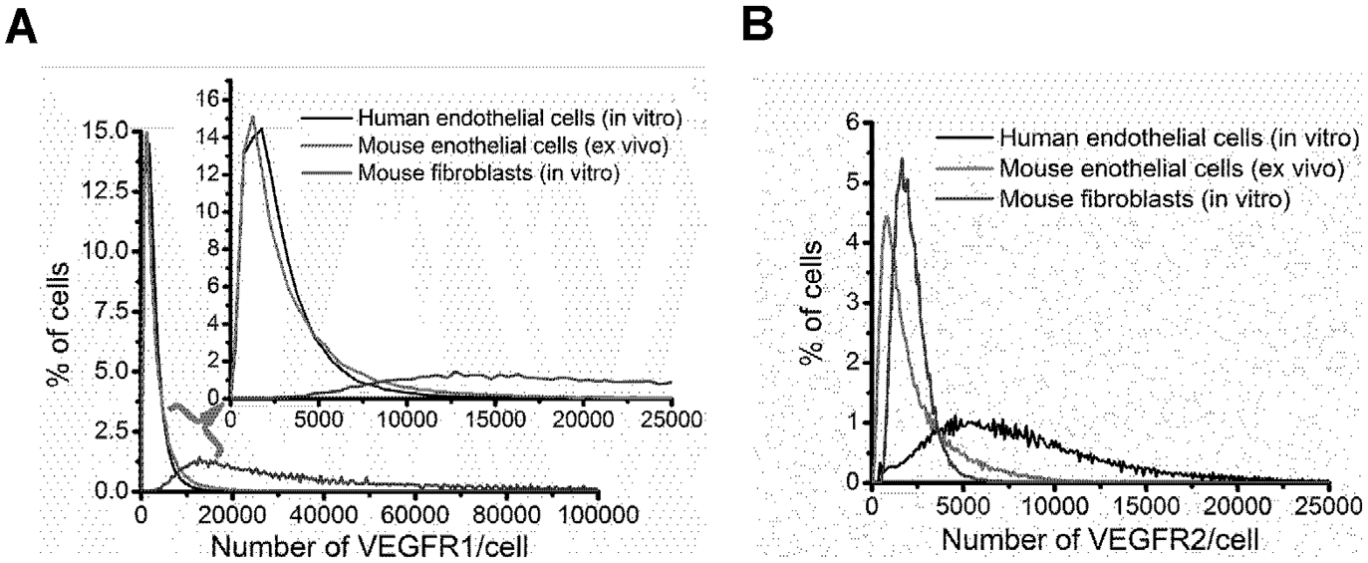
Cell-by-cell analysis of VEGFR1 and VEGFR2 distribution on ex vivo and in vitro cells. (A) Human and mouse endothelial cells display similar distributions of VEGFR1, the magnified traces show the distributions at the range encompassing a majority of the endothelial cells expressing VEGFR1, and it shows that the histograms overlap considerably, while the higher-VEGFR1-expressing fibroblasts have a much broader distribution of VEGFR1. (B) The histograms representing VEGFR2 levels on human in vitro endothelial cells, mouse ex vivo endothelial cells, and mouse in vitro fibroblasts have distinct profiles. The mouse endothelial cells have a large population expressing low levels of VEGFR2, and a broader range of cells expressing high levels of VEGFR2 relative to the mouse fibroblasts, which are more homogenous in their surface expression of VEGFR2. The human endothelial cells have much higher numbers of VEGFR2 on the cell surface, and the greatest heterogeneity in endothelial, VEGFR2 surface numbers.

Figure 3 displays the distributions of VEGFR surface localization on mouse endothelial cells. Qualitatively, the widths of the VEGFR1 and VEGFR2 histograms are similar; however, the consistently higher VEGFR1 mean, median, and mode, across each tissue and strain, compared to VEGFR2 underlie the difference in the distributions (Table 1, Figure 3). The higher VEGFR1 levels are also qualitatively described by the higher frequency of endothelial cells with high VEGFR1 following the density crossing point in each distribution (the point where the distributions intersect). A comparison of ex vivo mouse endothelial cells, in vitro mouse fibroblasts, and in vitro human endothelial cells allows greater comparison of the heterogeneity in VEGFR surface levels. Human in vitro endothelial cells and mouse ex vivo endothelial cells have similar VEGFR1 distributions; however, the lower mean and median VEGFR1 surface-levels on human ECs compared to mouse EC indicate a greater homogeneity in VEGFR1 surface-levels on the in vitro cells and greater ex vivo endothelial heterogeneity in VEGFR1 surface-levels (Figure 4). Qualitatively, ex vivo mouse endothelial cells have greater heterogeneity in VEGFR2 surface-localization compared to in vitro mouse fibroblasts, with in vitro human endothelial cells having the broadest distribution of VEGFR2 of the cell-types studied.

The cell-by-cell analysis reveals that there are statistically significant differences across strain and across muscle fiber with significantly higher VEGFR levels on BALB/c mice compared to C57Bl/6 mice and significantly higher VEGFR levels on tibialis anterior compared to gastrocnemius muscle. The cell-by-cell analysis also shows that the difference in mean densities of VEGFR1 and VEGFR2 is due to a slightly greater population of cells presenting low-numbers of VEGFR2. For example, in gastrocnemius muscle of BALB/c mice, 65% of endothelial cells have a VEGFR2 surface expression of less than 2,500 receptors/cell; whereas, ∼50% of endothelial cells have similar surface expression of VEGFR1 in the same mouse strain and tissue. This trend of low VEGFR2 surface expression is seen across each tissue and strain.

## Discussion

VEGFRs are the key elements in rendering the extracellular VEGF signal to an intracellular response. As such, their surface levels may significantly affect angiogenic signaling. Our results for the first time provide the range of VEGFRs found on endothelial cells within skeletal muscle and contextualize our findings through comparison to in vitro cell culture models. We show that VEGFRs are present at low levels under quiescent conditions, and we determine significant differences in the levels of VEGFR1 and VEGFR2.

Quantifying the ex vivo surface localization of angiogenic receptors, VEGFR1 and VEGFR2, can lead to a better understanding of the balance between pro-angiogenic, VEGFR2, and anti-angiogenic or modulatory, VEGFR1 signaling. Previous in vitro studies of VEGFR levels have reported VEGFR1 levels to be lower than VEGFR2 [12], [13], [14], [15], [17], [50]. The significantly higher surface presentation of VEGFR1 relative to VEGFR2 that we observe on ex vivo endothelial cells, presents a striking difference to the VEGFR1-VEGFR2 balance observed in monolayer endothelial cell culture. The differences between two-dimensional (2-D) culture systems and the native environment have been well studied in a number of cell types, including human mesenchymal stem cells, fibroblasts, smooth muscle cells, endothelial cells, and tumor cell lines [51], [52], [53], [54], [55], [56]. The absence of 3-D extracellular matrix interactions is a significant feature missing in monolayer cultures [53], [57], other differences include: the absence of mural cells [45], [58], [59] and the resulting growth factor supplementation in culture media [60], [61] and the absence of hemodynamic forces [62]. Altogether, these environmental differences can change cellular differentiation, proliferation, migration, and survival [63], and these factors possibly contribute to the inverted in vitro versus ex vivo VEGFR balance that we report. Future studies should systemically examine 3-D in vitro culture systems, mouse versus human endothelial cell cultures, and shear flow systems to identify if these parameters alter the VEGFR balance.

The average of 36,000 VEGFR1s per fibroblast, represents a significant number of VEGFRs, compared to the in vitro endothelial cell levels of 1,200–2,000 VEGFR1 per cell. The low-VEGFR2 levels we observe in these BALB/c derived fibroblasts correlates with previous studies showing no VEGFR2 protein expression in NIH3T3 fibroblasts [64], while the high-VEGFR1 expression on these BALB/c 3T3s differs from NIH3T3 fibroblasts, which do not natively express VEGFR1 [65], [66]. The high VEGFR1 surface-level raises the question of the role of VEGFR1 on this fibroblast cell line. Primary fibroblasts have previously been identified as expressing both VEGFR1 mRNA and protein with a functional role of inducing fibroblast migration [67]. In corneal fibroblasts, migration can be abolished with bevacizumab [68]. This VEGFR1 migratory function is also observed in macrophages [69], [70], monocytes [71], [72], [73], and endothelial cells [74]. The previous evidence of VEGFR1-mediated migration on fibroblasts, and the high levels of VEGFR1 we observe on BALB/c 3T3 fibroblasts warrants further study, and may present these cells as an in vitro model, in addition to NIH3T3-Flt-1 cells [65], [66], [69], for understanding VEGFR1 signaling dynamics.

The VEGFR surface-levels that we report are regulated by trafficking processes unique to each VEGFR. VEGF significantly regulates VEGFR2 trafficking by translocating endosomal-VEGFR2 to the plasma membrane [75] and to the nucleus [50]. VEGF also routes VEGFR2 to late endosomes and ultimately to degradation, generally via lysosomes and possibly through proteasomal mechanisms [76]. On average, we observe ∼1,600 VEGFR2 on the plasma membrane of endothelial cells isolated from skeletal muscle. Trafficking studies reveal a significant, but lesser fraction, 40% of total VEGFR2 or ∼1,000 VEGFR2 by our estimate, residing intracellularly [77], [78] in endosomal storage compartments, [75], [76], [79], [80], [81]. A portion of those intracellular VEGFR2 stores, ∼50% or ∼500 VEGFR2 by our estimate, are then constitutively recycled. These estimates of ∼1,000 intracellular VEGFR2/cell and ∼500 constitutively recycling VEGFR2/cell assume that in vitro sub-cellular partitioning applies to ex vivo cells [78]; future studies should examine VEGFR trafficking in ex vivo or in vivo systems. In contrast, VEGFR1, which we find at higher levels on the cell surface compared to VEGFR2 (∼2,800 VEGFR1/endothelial cell) (Table 1), is predominantly localized intracellularly. By our estimate, intracellular VEGFR1 constitute ∼11,000 receptors/cell based on an previous studies showing ∼80% of total VEGFR1 being intracellular in the Golgi apparatus [77] and in the nucleus [50], and VEGFR1 shows little to no constitutive recycling [82]. VEGFR1 levels can also be regulated by VEGF, which stimulates trans-Golgi network-to-plasma membrane translocation of VEGFR1 [77], [83].

Connecting the abundance of VEGFRs on the cell-surface with VEGFR intracellular partitioning provides new context for the role of intracellular VEGFR signaling in angiogenesis. Studies of VEGFR2 association with vascular endothelial (VE) cadherin have shown increased VEGFR2 internalization and sustained phosphorylation of VEGFR2 in the absence of VE-cadherin, suggesting the presence of VEGFR2 intracellular signaling [80]. Intracellular tyrosine kinase receptor signaling has also been established through studies of EGFR endosomal accumulation. Those studies identified cell surface EGFRs as promoting cell growth and intracellular EGFRs as inducing apoptosis in MDA-MB-468, breast cancer cells [84]. Other studies have identified the presence of extracellular signal-regulated kinase (ERK)-mitogen-activated protein kinase (MAPK) components on EGFR-containing endosomes, and the presence of ligand-bound EGFRs on endosomes, suggesting the occurrence of EGFR endosomal signaling [85], [86]. In light of these previously published data and our intracellular VEGFR estimates, future studies should further explore the contribution of intracellular VEGFR signaling on endothelial cell proliferation and migration: determining any endosomal complexes regulating signaling outcomes and identifying whether intracellular signaling occurs through differential cellular pathways.

Angiogenic signaling is complicated by the homo- and hetero-dimerization of VEGFRs. Recently, VEGF mediated dimerization of VEGFR2/3, VEGFR2/2, & VEGFR3/3 was reported in human saphenous endothelial cells, displaying a role for VEGFR2 & VEGFR3 (pro-lymphangiogenic receptors) dimerization in regulating angiogenic sprouts [87]. Homodimers VEGFR2/2 display pro-angiogenic signaling, while heterodimers VEGFR1/2 are not only functional but may affect pro-angiogenic signaling through VEGFR2 [88], [89], [90]. Previous computational modeling of in vitro VEGFR1 and VEGFR2 dimerization in the presence of picomolar to nanomolar VEGF, predicted the percentage of heterodimeric VEGFR1/2 complexes to be ∼30–50%, homodimeric VEGFR1/1 complexes to be 65–30%, and VEGFR2/2 complexes to be ∼5–20%, when the number of VEGFR1 on the cell surface equals the number of VEGFR2 [11]. Furthermore, when the ratio of VEGFR1 to VEGFR2 increases, the number of VEGFR1/1 homodimers significantly increases (up to 85–96% at a 10∶1 VEGFR1:VEGFR2 ratio). The mouse endothelial cells examined in our study display approximately 2∶1 ratio of VEGFR1:VEGFR2, suggesting that a significant fraction of the dimerized receptors would contain VEGFR1 (>95% based on the above estimates). These dimerization data coupled with our quantification provide a putative mechanism for the role of VEGFR1 in mouse in vitro fibroblasts and mouse ex vivo endothelial cells, and a mechanism for dominating VEGFR2 signaling in in vitro human endothelial cells. These data also demonstrate a need to establish the signal response of VEGFR1/2 heterodimers.

A challenge in the emerging field of personalized medicine is identifying variability in patient populations, which can result in differential therapeutic outcomes [25]. It has been proposed that differences in VEGF expression, vascular density and responses to ischemia observed across the C57BL/6 and BALB/c mouse strains [19], [21], [28] may lend these mice as a proxy for human population variability [25]. Our results show small, but significant differences in VEGFR levels across these strains. A significant difference in VEGFR localization was also seen across fiber types, with higher VEGFR1 surface-localization in the glycolytic tibialis anterior muscle compared to the mixed gastrocnemius muscle. Hallmarks of glycolytic muscle include its lower capillary density [91], lower capillary to fiber ratio [92], and lower VEGF mRNA levels compared to oxidative muscle [29], [30]. These previously determined differences in vascular properties across fiber types may contribute to the differences in VEGFR1 surface levels, which we observe.

Cell by cell analysis shows that most of the mouse endothelial cells (>95%) have a surface localization between 0–12,000 VEGFRs/cell, representing significant heterogeneity in the number of VEGFRs that can be expressed by mouse endothelial cells. A comparison of mouse ex vivo and in vitro and human in vitro VEGFR distributions contextualizes this heterogeneity. The lower VEGFR1 dispersion on mouse endothelial cells relative to mouse fibroblasts suggests greater control of VEGFR1 signaling on the endothelial cells. Similarly, the lower VEGFR2 dispersion on the mouse endothelial cells relative to the human endothelial cells suggests greater signaling control within the ex vivo mouse system. The basis of endothelial-VEGFR heterogeneity may be functional, such as the presence of endothelial sub-populations (e.g., tip versus stalk cells, arteriolar versus venular capillary side) and conditional, such as genomic, proteomic, or environmental effectors resulting in differential VEGFR presentation at the cell surface [35], [93]. Systems biology methods can be applied to identify the significance of functional variability in VEGFR expression and signaling, as demonstrated by agent-based modeling determining the role of VEGF and delta-like 4 (Dll4)/notch in endothelial tip cell selection [94], [95]. Furthermore, advancing genomic, proteomic, and environmental modeling can further elucidate the significance of these factors in VEGFR signaling.

### Implications of ex vivo Quantitative Flow Cytometry

Our laboratory has developed whole-body models of VEGF-VEGFR binding kinetics [9], [96], which have predicted the distribution of VEGF in the body upon administration of the anti-VEGF antibody, bevacizumab, delineating the mechanism of action of this therapeutic agent [96], [97]. Such models have the power to predict the optimal drug and tumor properties for which an anti-VEGF agent may have an advantageous effect. However, the predictive power of these models has previously been limited by insufficient knowledge of cell surface receptor densities. Therefore, the data we report provide critical parameters needed to advance angiogenesis models [97], [98], [99], [100], [101], [102], [103], [104].

These quantitative approaches can be further extended to understand angiogenic signaling. Many angiogenic receptors [105], [106], [107], [108], [109], including the VEGFRs are known to reside on lipid rafts or localize on caveolin-rich microdomains [81], [110], [111], [112], [113], [114], with possible functional roles of compartmentalizing, amplifying, and coordinating signaling and even regulating receptor trafficking [115], [116], [117]. Therefore, the number of proteins on these rafts may significantly affect angiogenic signaling [117], [118], [119]. Recent studies of platelet-derived growth factor receptors (PDGFR) have shown that non-rafted receptors can be differentially internalized and preferentially activated compared to raft-localized PDGFRs [115]. If rafts similarly affect VEGFRs, then it will become important to further determine VEGFRs distribution on and off lipid rafts. The ex vivo quantitative approaches described, here in combination with microscopy and systems biology methods can be further extended and to sensitively measure angiogenic raft composition and its effect on signaling. This is the first study to quantitatively characterize angiogenic receptor localization, ex vivo. We present a framework for interpreting the complex cues within the vascular microenvironment through the coupling of endothelial cell isolation with cell-by-cell mapping of angiogenic receptor levels. Furthermore, this quantitative approach can be used to profile receptor levels in vascular pathologies. Our research team is currently merging this quantitative approach with computational modeling to inform on the cellular heterogeneity in cancer and ischemic disease that can contribute to differential therapeutic outcomes on the patient level. Altogether, these approaches and these data provide a fundamental understanding of VEGFRs within the quiescent vascular microenvironment.

## Supporting Information

Figure S1 **Representative flow cytometry plots for a total cell suspension, which would include all components of skeletal muscle such as: myocytes, endothelial cells, pericytes, etc (A) forward scatter versus side scatter plot for raw cell suspension.** (B–E) single-cell gated FITC versus PE plots for (B) non-labeled cells, (C) anti-CD34-FITC labeled cells, (D) anti-VEGFR2-PE labeled cells, and (E) both anti-CD34-FITC and anti-VEGFR2-PE labeled cells. The CD34+/VEGFR2+ cells represent ∼5% of the total cell population.
(PDF)

Figure S2 **Representative forward scatter and side scatter plots for (A) human umbilical vein endothelial cells (HUVEC), (B) mouse skeletal muscle endothelial cells (SkM), (C) fibroblasts (3T3), and (D) PE beads**
(PDF)

Figure S3 **Representative FL1 (FITC) versus FL2 (PE) plots for (A) non-labeled HUVECs, (B) HUVECs labeled with anti-CD31-FITC, (C) HUVECs labeled with anti-VEGFR2-PE, and (D) HUVECs labeled with both anti-CD31-FITC and anti-VEGFR2-PE.**
(PDF)

Figure S4 **Representative FL1 (FITC) versus FL2 (PE) plots for (A) non-labeled SkM, (B) SkM labeled with anti-CD34-FITC, (C) SkM labeled with anti-VEGFR2-PE, and (D) SkM labeled with both anti-CD34-FITC and anti-VEGFR2-PE.**
(PDF)

Figure S5 **Representative FL1 (FITC) versus FL2 (PE) plots for (A) non-labeled 3T3, (B) 3T3 labeled with anti-VEGFR1-PE.**
(PDF)

Figure S6 **Representative PE bead (A) histogram and (B) calibration curve.**
(PDF)

Figure S7 **Cell surface expression of (A) VEGFR1 and (B) VEGFR2 in male and female mice.**
(PDF)
