# Supplementary figures and images for: Expression of VEGF Receptors on Endothelial Cells in Mouse Skeletal Muscle
Source: PLoS One. 2012 Sep 12;7(9):e44791. doi: 10.1371/journal.pone.0044791 (PMC3440347; doi:10.1371/journal.pone.0044791)

**A**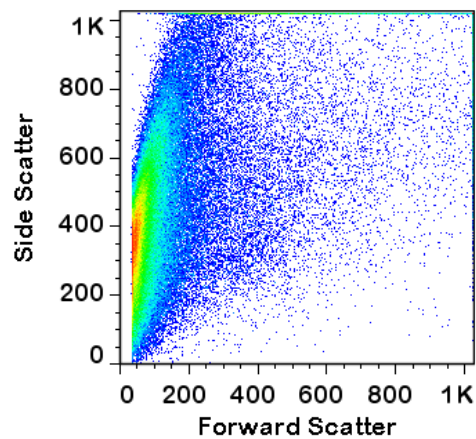**B**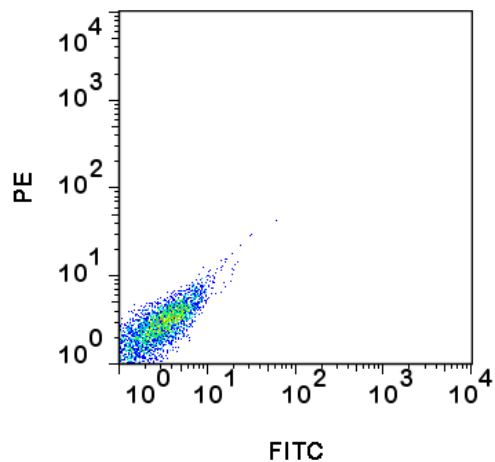**C**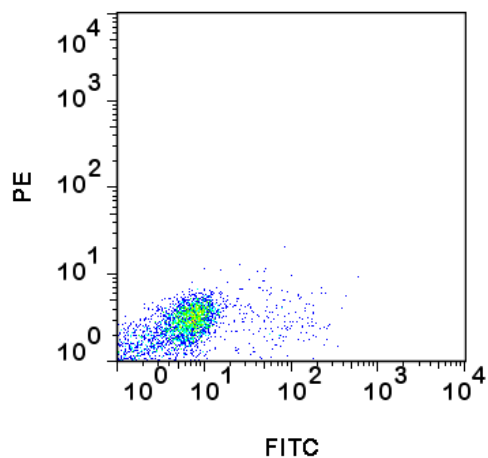**D**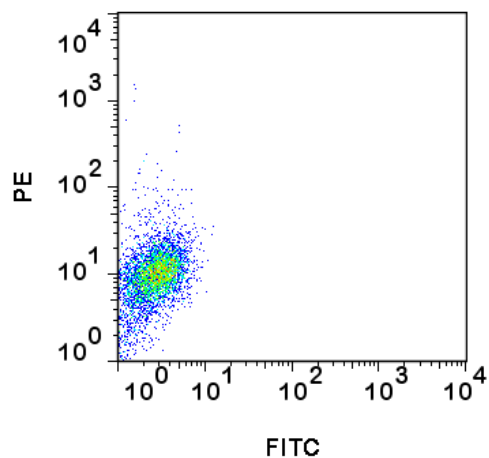**E**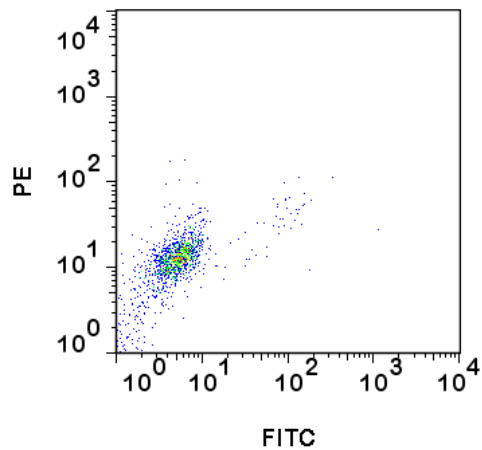**Figure S1**

Supplement: Figure S1 — Representative flow cytometry plots for a total cell suspension, which would include all components of skeletal muscle such as: myocytes, endothelial cells, pericytes, etc (A) forward scatter versus side scatter plot for raw cell suspension. (B–E) single-cell gated FITC versus PE plots for (B) non-labeled cells, (C) anti-CD34-FITC labeled cells, (D) anti-VEGFR2-PE labeled cells, and (E) both anti-CD34-FITC and anti-VEGFR2-PE labeled cells. The CD34+/VEGFR2+ cells represent ∼5% of the total cell population. (PDF) [file pone.0044791.s001.pdf]

**A**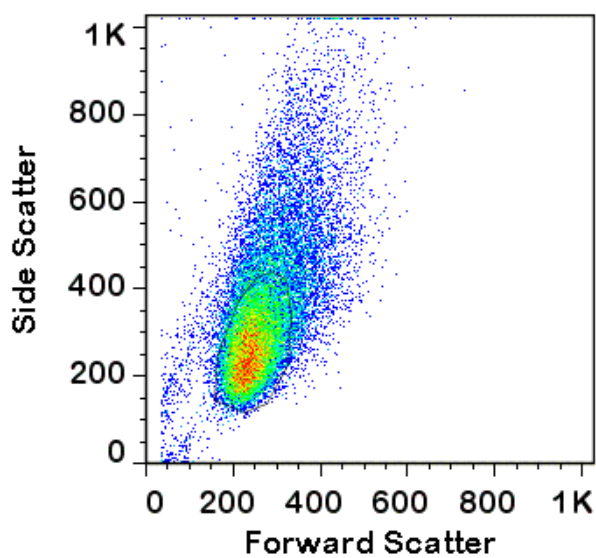**B**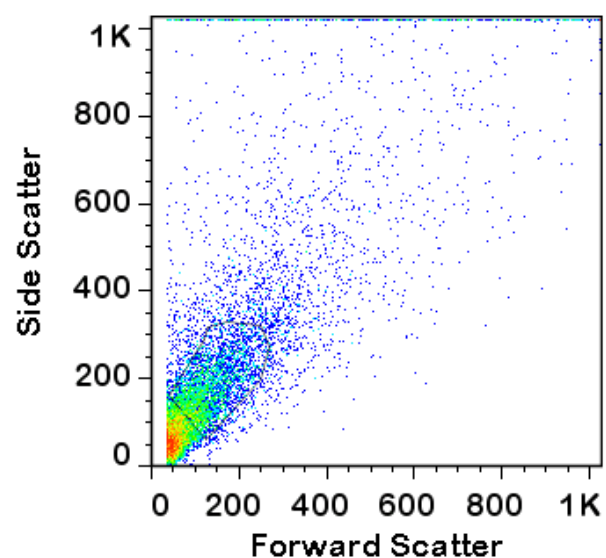**C**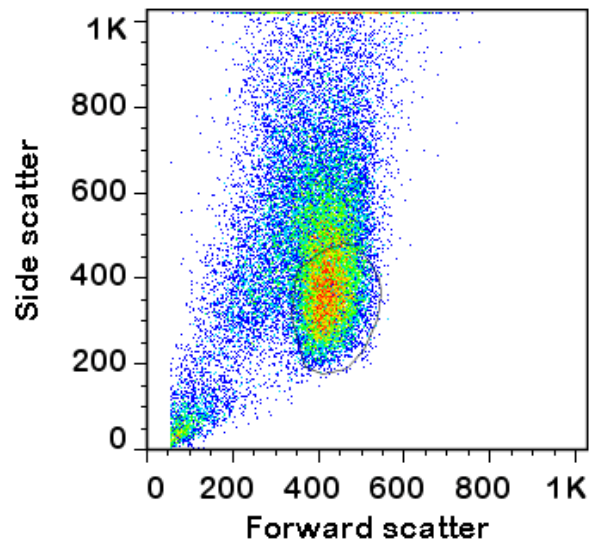**D**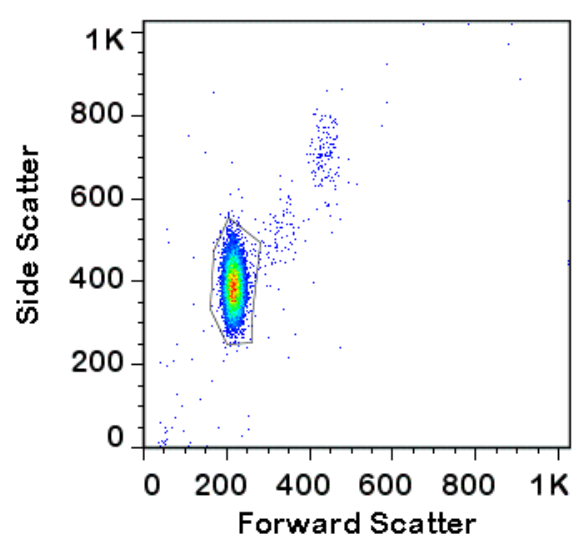

**Figure S2**

Supplement: Figure S2 — Representative forward scatter and side scatter plots for (A) human umbilical vein endothelial cells (HUVEC), (B) mouse skeletal muscle endothelial cells (SkM), (C) fibroblasts (3T3), and (D) PE beads (PDF) [file pone.0044791.s002.pdf]

**A**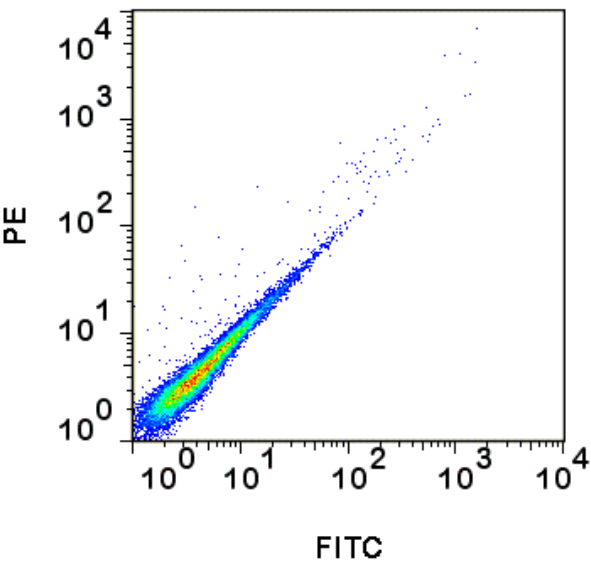**B**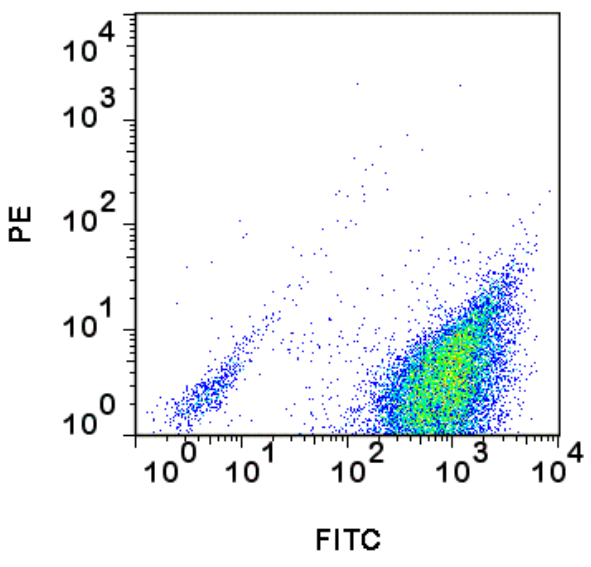**C**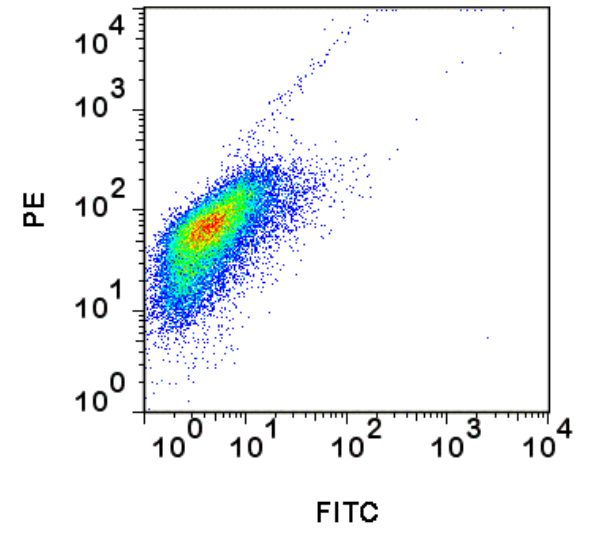**D**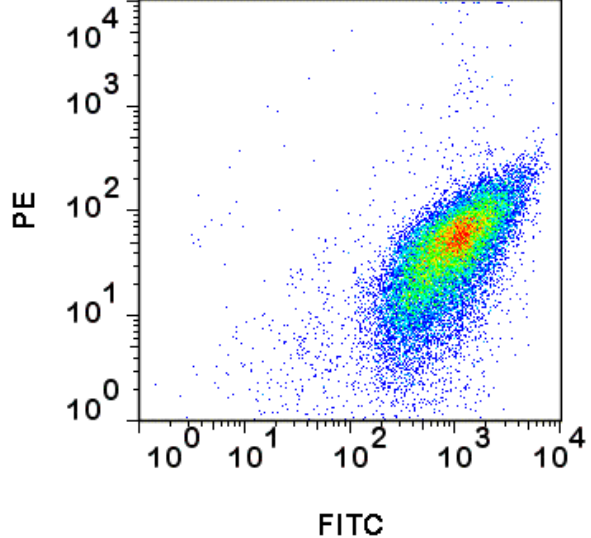

**Figure S3**

Supplement: Figure S3 — Representative FL1 (FITC) versus FL2 (PE) plots for (A) non-labeled HUVECs, (B) HUVECs labeled with anti-CD31-FITC, (C) HUVECs labeled with anti-VEGFR2-PE, and (D) HUVECs labeled with both anti-CD31-FITC and anti-VEGFR2-PE. (PDF) [file pone.0044791.s003.pdf]

**A**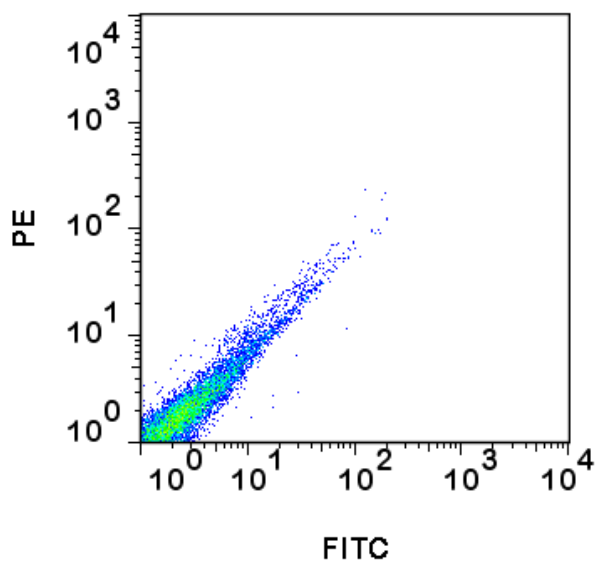**B**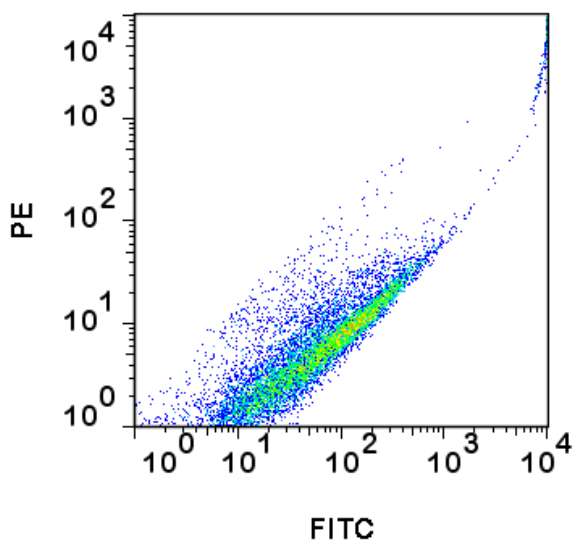**C**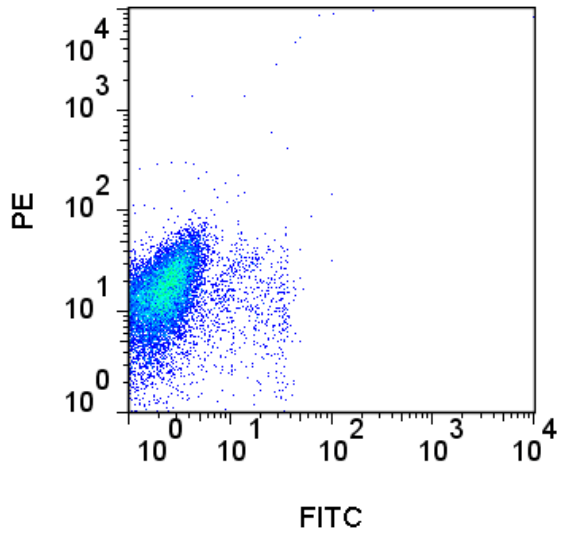**D**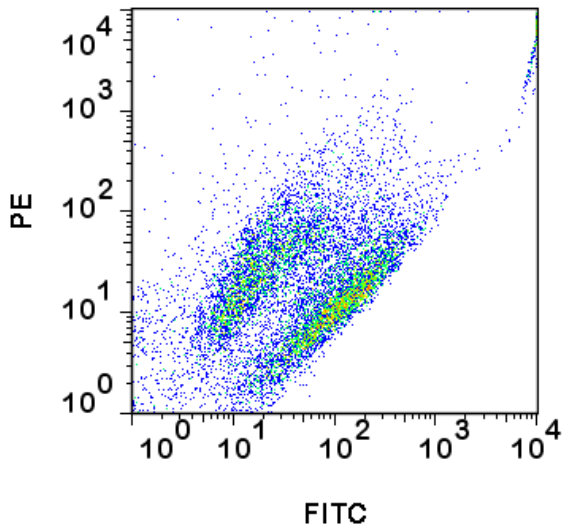

**Figure S4**

Supplement: Figure S4 — Representative FL1 (FITC) versus FL2 (PE) plots for (A) non-labeled SkM, (B) SkM labeled with anti-CD34-FITC, (C) SkM labeled with anti-VEGFR2-PE, and (D) SkM labeled with both anti-CD34-FITC and anti-VEGFR2-PE. (PDF) [file pone.0044791.s004.pdf]

**A**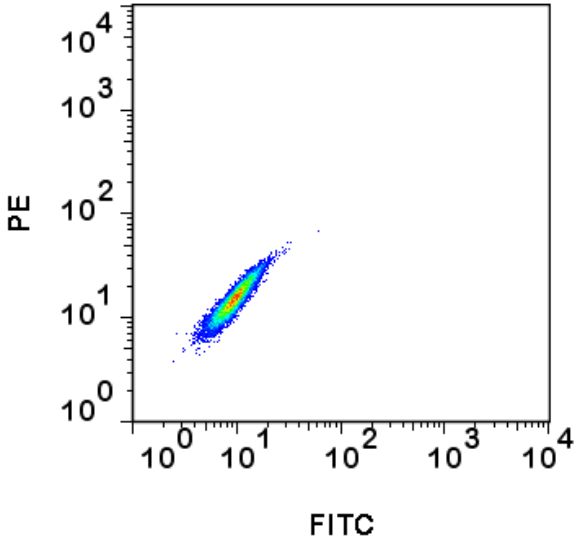**B**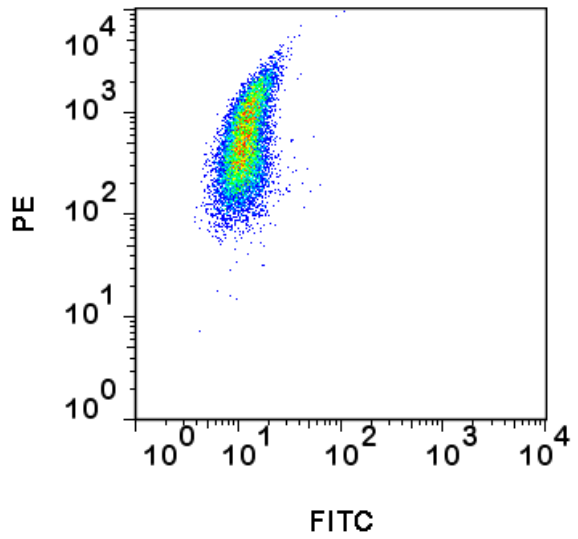

**Figure S5**

Supplement: Figure S5 — Representative FL1 (FITC) versus FL2 (PE) plots for (A) non-labeled 3T3, (B) 3T3 labeled with anti-VEGFR1-PE. (PDF) [file pone.0044791.s005.pdf]

**A**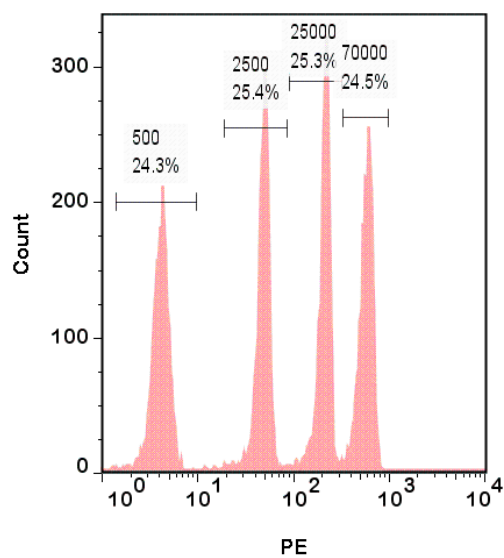**B**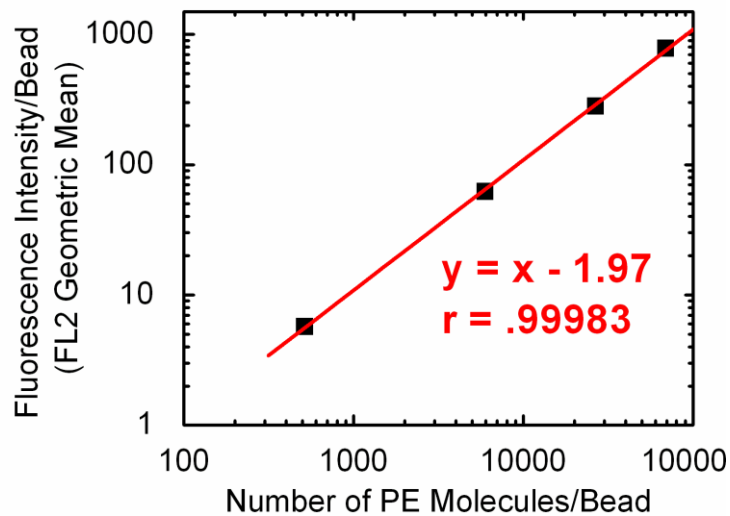**Figure S6**

Supplement: Figure S6 — Representative PE bead (A) histogram and (B) calibration curve. (PDF) [file pone.0044791.s006.pdf]

**A**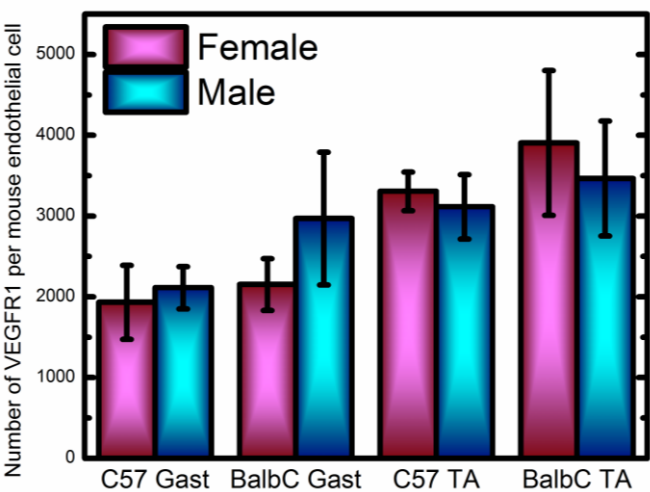**B**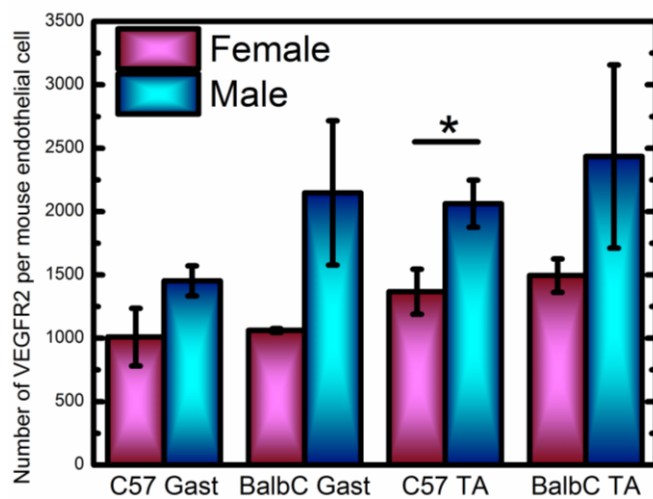**Figure S7**

Supplement: Figure S7 — Cell surface expression of (A) VEGFR1 and (B) VEGFR2 in male and female mice. (PDF) [file pone.0044791.s007.pdf]
